# Future perspective of stem cell-derived exosomes: Cell-free therapeutic strategies for retinal degeneration

**DOI:** 10.3389/fbioe.2022.905516

**Published:** 2022-11-14

**Authors:** Zibin Liu, Fang Zeng, Yao Zhang, Yongqing Liu, Zhuo Li, Xiao Liu

**Affiliations:** ^1^ Department of Ophthalmology, The Second Xiangya Hospital, Central South University, Changsha, China; ^2^ Hunan Clinical Research Center of Ophthalmic Disease, Changsha, China; ^3^ Department of Neurology, Hunan Provincial People’s Hospital, Hunan Normal University, Changsha, China; ^4^ Department of Medicine, University of Louisville School of Medicine, Louisville, KY, United States

**Keywords:** exosomes, stem cell-derived exosomes, stem cell, retinal degeneration, cell-free therapy

## Abstract

With continued expansion of the aged population, the number of patients with retinal degeneration, which is a leading cause of vision loss worldwide, is growing. Stem cell therapies offer hope for regeneration and repair of damaged retinal tissue. Recent reports have highlighted stem cell-derived paracrine mediators, such as exosomes, which appear to exert a therapeutic benefit similar to their cell of origin and do not carry the risk of cell transplantation. One speculated role is that exosomes likely mediate intercellular communication and material exchange. This review depicts the molecular mechanisms underlying exosome-based therapy, especially in retina degeneration diseases. In the future, the use of stem cell-derived exosomes could be considered a novel and cell-free therapeutic strategy in regenerative medicine.

## 1 Introduction

### 1.1 Retinal degeneration (RD)

The retina, an essential part of the eyeball, is composed of multiple kinds of neurons, including photoreceptor, bipolar, horizontal, amacrine, and ganglion cells, located right before the retinal pigment epithelium (RPE), a single layer of epithelial cells. Externally, the RPE closely adheres to the choroid in the back and faces the outer segment of the light-perceiving photoreceptors in the front. Its apical and basal sides can secrete several different growth factors, which not only nourish the photoreceptor cells but also phagocytize the outer segment debris.

The RPE is a vital anatomical structure in the eye with irreplaceable properties that maintains key functions of the retina. As such, any defect in the mammalian RPE may lead to RD as a reduction in the supply of nutrients from the RPE can cause the photoreceptors to die of starvation. As these fully differentiated cells are not able to proliferate to replace defective cells after birth, it is therefore extremely difficult to mediate retinal regeneration (Strauss, 2005; Campbell and Humphries, 2013; Stern et al., 2018; Kwon and Freeman, 2020; Shen, 2020). RD can be divided into two major types: age-related macular degeneration and inherited retinal dystrophy (Hamel, 2006; Hohman, 2017; Gagliardi et al., 2019).

### 1.2 Stem cells and stem cell therapy

Over many years, the definition of stem cells has been revised according to different perspectives. Based on their potential, stem cells are divided into totipotent stem cells (zygote or the fertilized egg), pluripotent stem cells (PSCs), multipotent stem cells, oligopotent stem cells, and unipotent stem cells (also known as progenitor cells). Totipotent stem cells can produce all somatic cells, giving rise to both the placenta and the embryo. Zygotes are the only type of totipotent stem cells. PSCs include both embryonic stem cells (ESCs) and induced PSCs (iPSCs). ESCs are derived from the inner mass of the early embryo (the blastocyst) (4–5 days); possess pluripotency; and can differentiate into ectoderm, mesoderm, and endoderm germ layers. They were first isolated by Thomson et al. (1998). iPSCs were first reported by Kazutoshi Takahashi and Shinya Yamanaka in 2006 (Takahashi and Yamanaka, 2006), are artificially created by introducing four essential stem cell genes into fibroblasts using a technology called cell reprogramming, and have stem cell properties similar to those of ESCs.

Multipotent stem cells can generate multiple cell types within a specific tissue, such as mesenchymal stem cells (MSCs), cardiac stem cells, or hematopoietic stem cells, or are derived from connective tissue and the interstitium of organs, especially bone marrow. Moreover, oligopotent stem cells are similar to MSCs with less potential to give rise to ≥2 types of cells within a tissue, such as neural stem cells. In contrast, unipotent stem cells can only give rise to a single type of cell, such as spermatogonial progenitor stem cells (Bosio et al., 2011).

Many studies have focused on the treatment of RD, assessing the usage of neurotrophic factors, gene therapy, retinal transplantation, stem cells, or drugs (Sieving et al., 2006; Gasparini et al., 2019; Vazquez-Dominguez et al., 2019). Stem cell therapy has been favored due to these cells’ self-renewal ability and potential to evolve into a variety of cell types (Bosio et al., 2011). Normally, the underlying mechanism of the stem cell therapeutic effect is regarded as the differentiation and replacement of the lost and/or defected cells to ultimately restore or rescue the function of the affected tissue. However, further experiments have revealed that the transplanted stem cells can face a much more complex and evolving situation than expected when differentiating into the exact right cell type(s) *in vivo*. Even if they develop into cells of the right lineage(s), they may not survive long enough to properly function in the grafted site due to the unfriendly and uncertain microenvironment in the diseased tissue, which may be characterized by oxygen deficiency, oxidative stress, inflammation, and other immune response. As such, the therapeutic effects of the grafted cells often last only a few days after transplantation. In such a short time, it is hard for the grafted stem cells to differentiate into the targeted cells and establish a functional relationship with their surrounding neighbors (Prockop, 2007; Najar et al., 2020). In addition, MSCs can exert their regenerative and immunomodulatory effects without migrating to the right damaged tissue, suggesting that the therapeutic effect is not fully realized due to cell replacement (Klingeborn et al., 2018; Stahl and Raposo, 2018; Najar et al., 2020). Although functional integration of transplanted cells is key for the success of cell-replacement therapies, it is not necessarily required for cell remediation (Prockop, 2007; Najar et al., 2020). Montemurro et al. (Montemurro et al., 2016) investigated the relationship between the angiogenic and anti-inflammatory properties of MSCs collected from cord blood for cell regeneration and found that the secreted factors and extracellular vesicles (EVs) are the vital elements of the MSC-conditioned media (MSC-CM). Liu et al. (2010) studied the connection between MSC-CM and neuron injury induced by ethanol and revealed that MSC-CM can prevent ethanol-induced chronic oxidative damage by reducing reactive oxygen species. Mahmoudi et al. (2019) researched the protective effects of MSC-exosomes (MSC-Exos) and other substances in MSC-CM on neutrophils and found that they could enhance the ability of neutrophils to resist pathogens. Further, Linero and Chaparro et al. (2014) and Rota et al. (2018) corroborated the finding that the existence of a more positively protective and regeneration effect of the MSC actually occurs through MSC-CM.

**FIGURE 1:**
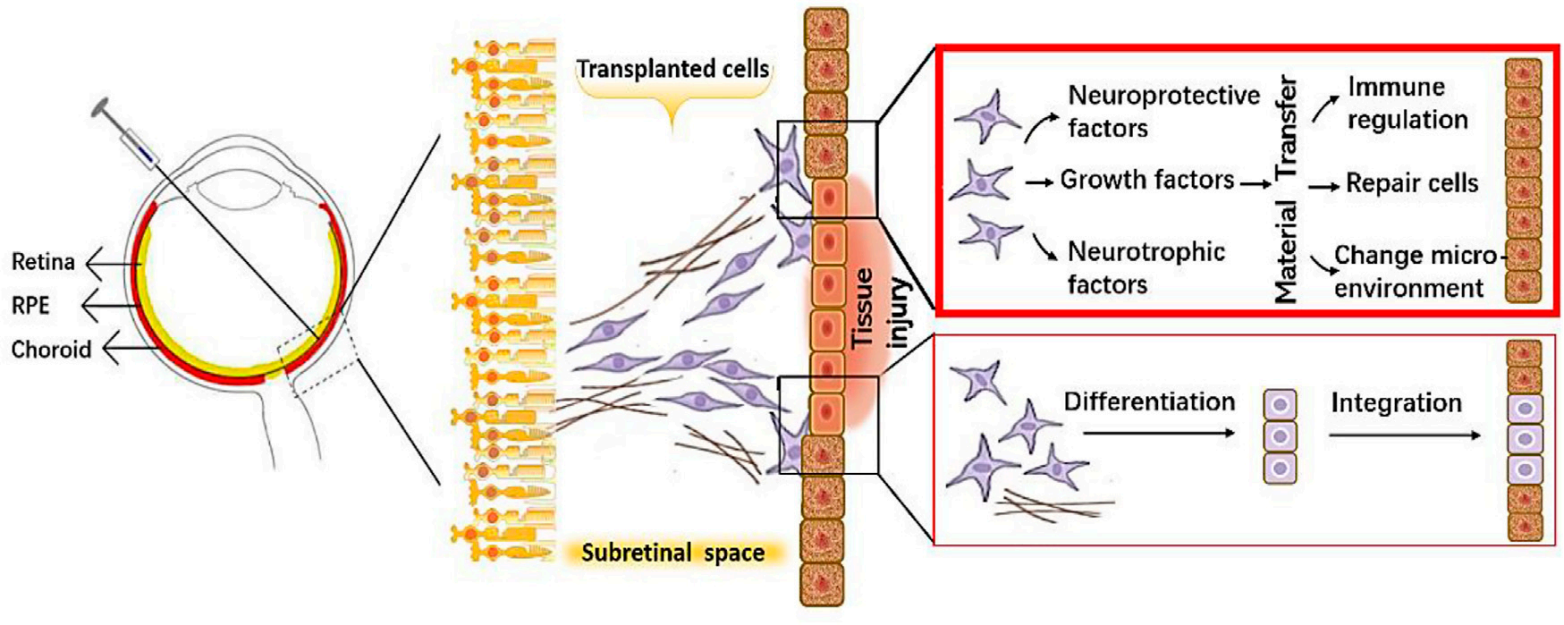
A schematic diagram showing protective mechanisms of the transplanted cells on retinal cells.

The same verdicts have been rendered in research on RD (Klingeborn et al., 2018; Stahl and Raposo, 2018). Therefore, the realization has been made that it is not the process of cell replacement as initially expected that is key to therapeutic success but instead the substances that the grafted cells secrete at the affected site—especially the EVs, which promote material transfer and protect and repair the retinal tissue through modulation of the immune response, inhibition of fibrosis, promotion of angiogenesis, and diminution of cell apoptosis (Figure 1).

### 1.3 Therapeutic effect of stem cell-derived EVs in ophthalmic diseases

#### 1.3.1 Paracrine action and extracellular vesicles

The action wherein cells secrete substances to communicate with other cells is known as paracrine action. In addition to soluble substances, cells are also known to secret plasma membrane–wrapped substances, collectively referred to EVs (Andaloussi et al., 2013). EVs are roughly classifiable into ectosomes and exosomes. Ectosomes bud out directly from the plasma membrane and are grouped based on their vesicle size into macrovesicles, microparticles, and large vesicles. Their size can range from 50 to 1,000 nm in diameter (Gould and Raposo, 2013; van Niel et al., 2018). Meanwhile, exosomes originate from the endosomal system (Kalluri and LeBleu, 2020), with a possible diameter of 30–150 nm (Gould and Raposo, 2013; S et al., 2013; van Niel et al., 2018), and they are enclosed by a lipid bilayer. The plasma membrane initially invaginates to form an early-sorting endosome, with subsequent maturation into a late-sorting endosome. The membrane invagination of a late-sorting endosome (the second time) then leads to the generation of intracellular multivesicular bodies that fuse with the cell membrane and release the intraluminal vesicles they contain to form exosomes (van Niel et al., 2018). This process is part of the intracellular plasma membrane circulation (van Niel et al., 2018). The exosomes contain multiple proteins, lipids, messenger RNAs (mRNAs), and microRNAs (miRNAs) that are not only rich in macromolecules inside the vesicles but also exist with distinctive marks on the surface for being marked and tracked.

Different protein markers can be expressed on the surface of an exosome depending on the cell type from which it was derived. The EV-packaging process has ≥2 different ways of packing EVs, such as the Endosomal Sorting Complex Required for Transport (ESCRT) complex–dependent pathway and the ESCRT complex–independent pathway. Therefore, the contents of exosomes, even if secreted by the same cell, can be distinct. With a microenvironmental change in the parent cells, such as with exposure to hypoxic conditions or intervention with certain medicines, the exosome contents can be altered accordingly to reflect the state of the parent cells and the secretion and cargo of EVs from tumor cells could change (Jafari et al., 2020; Soraya et al., 2021). Moreover, exosomes may exert different biological effects on the targeted cells in a manner that hinges on their surface receptors (Mathivanan et al., 2012; Cai et al., 2020).

**FIGURE 2:**
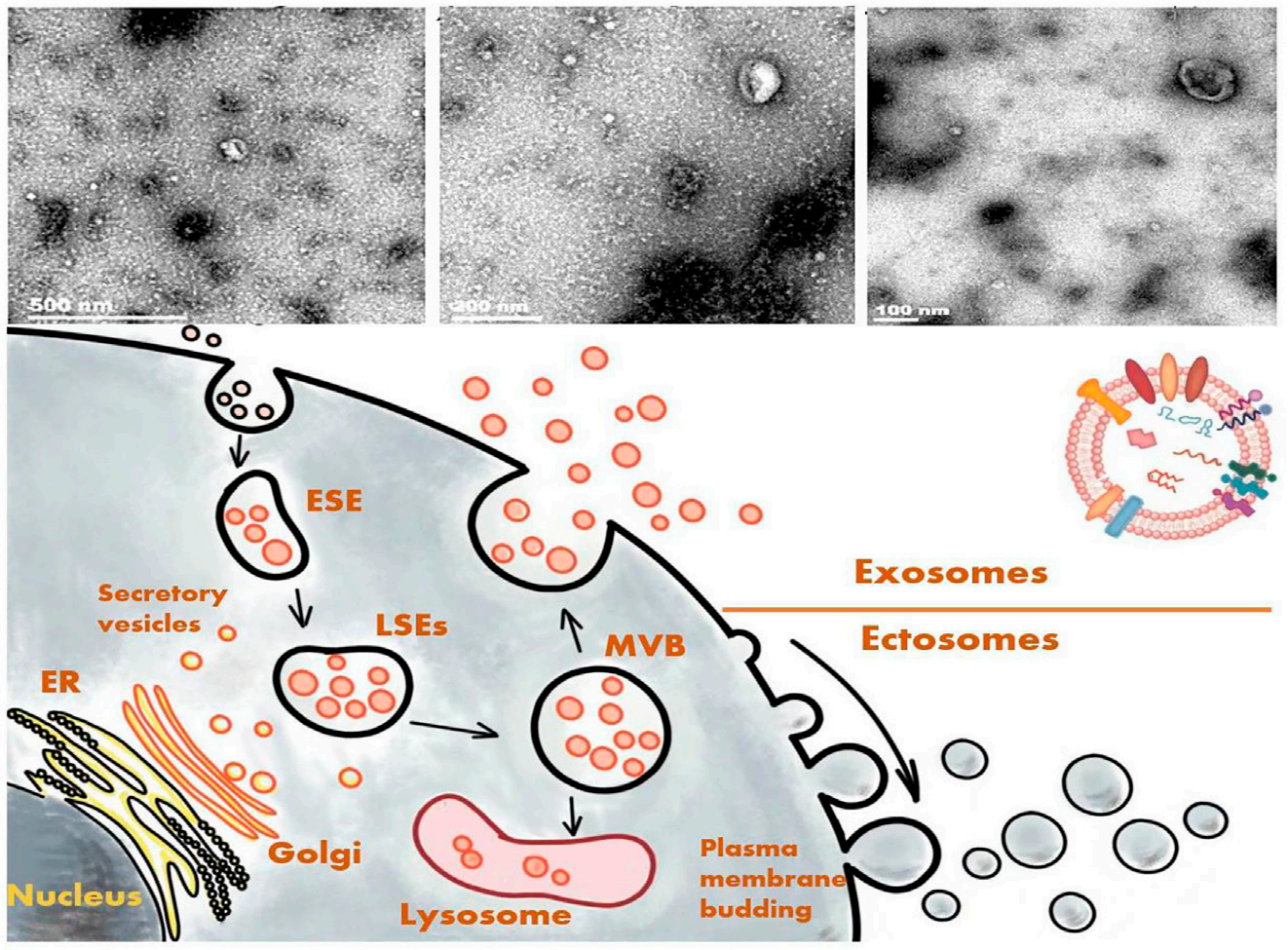
Formation process and electron microscope images of exosomes.

As mentioned above, exosomes have some unique macromolecular markers, providing specific traits for their differentiation from other substances. Among them, one of the most typical characters is the tetraspanin, a member of the transmembrane protein family consisting of four transmembrane domains with >30 proteins (Hemler, 2003), such as cluster of differentiation (CD)9, CD63, CD81, and CD82. According to their endosomal origin, some membrane transporters and fusion proteins, such as flotilin and the ras-related protein rab5b or Alix, which are all involved in the biogenesis of multivesicular bodies and the tumor-susceptibility gene 101 protein, can serve as markers of exosomes (Vlassov et al., 2012; Vitha et al., 2019) (Figure 2). Because exosomes and macrovesicles overlap in diameter, according to the 2014 statement of the International Society for Extracellular Vesicles (ISEV), both can be collectively referred to as EVs. ISEV acknowledged that researchers often used “exosomes” to refer to any EVs (Lötvall et al., 2014; Stahl and Raposo, 2018). We as the authors of this review will adopt the same approach based on the classification of the references.

In previous studies on the composition and nature of exosomes, the milestone event was undoubtedly the discovery that exosomes could contain both messenger RNA and miRNA at the same time (Kowal et al., 2014; Kalluri and LeBleu, 2020). Further, EVs not only adhere to nearby cells but also travel through body fluids to affect distant cells, and exosomes can interact with targeted cells by directly activating cell surface receptors *via* protein and bioactive lipid ligands, releasing signaling factors, or through endocytosis (Ratajczak et al., 2006; Valadi et al., 2007). Exosomes are now considered to be involved in several biological processes, such as immune response, viral pathogenicity, intercellular communication, and the progression of diseases (Jodo et al., 2001; Valadi et al., 2007; Théry et al., 2009; Wang et al., 2011).

#### 1.3.2 Stem cell-derived EVs in the treatment of ophthalmic diseases

**FIGURE 3:**
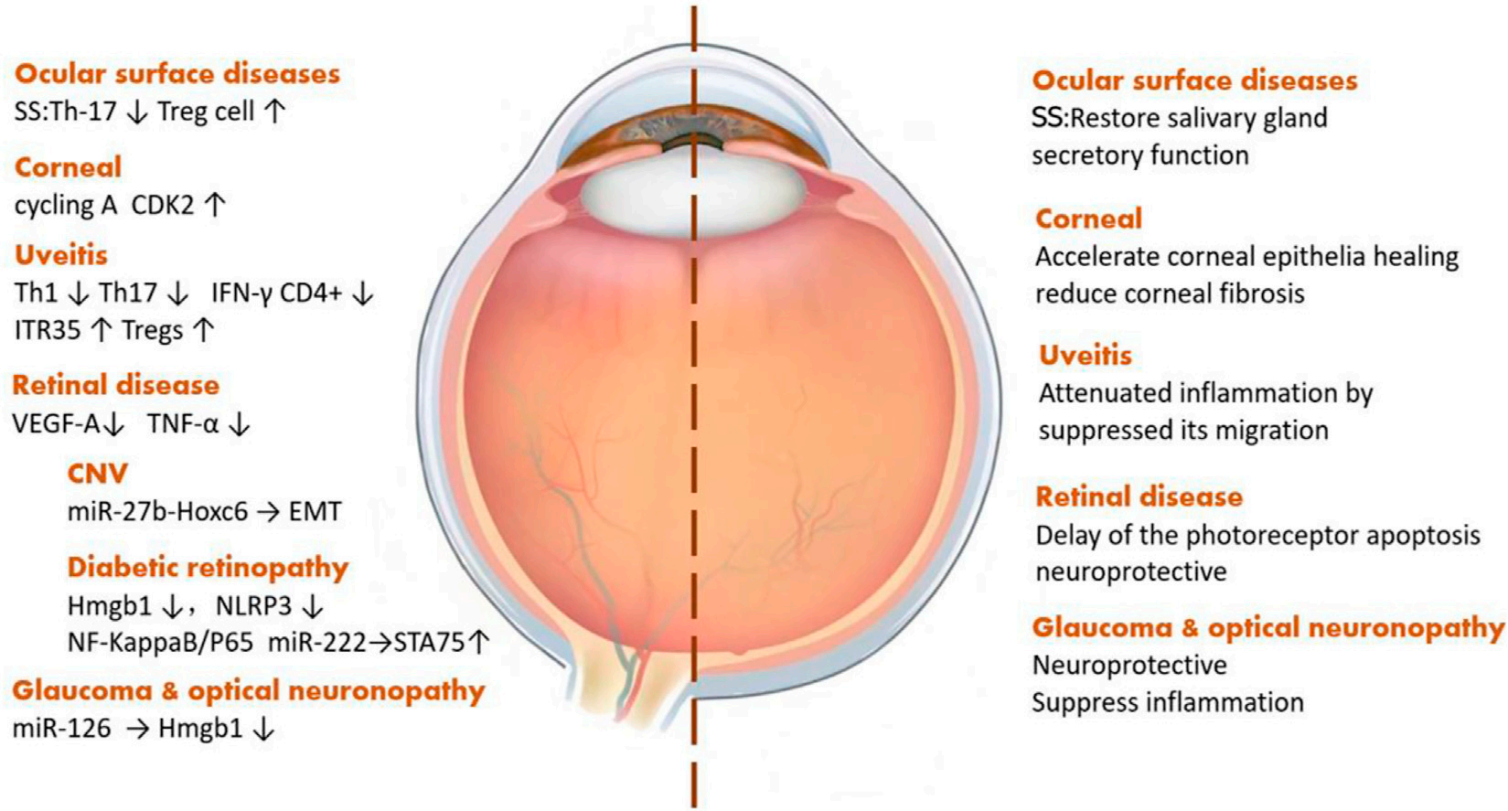
Exosomes derived from stem cells play different roles in ophthalmic diseases.

Exosomes derived from stem cells play a vital role in a variety of ophthalmic diseases, such as ocular surface diseases, uveitis, and optic neuropathy. Sjögren’s syndrome, a kind of systemic disease related to the presence of exocrine gland lesions on several organs, like the lacrimal glands and salivary glands, can affect lacrimal gland secretion, causing dry eye and wrecking the ocular surface health. MSC-Exos can restore gland secretion by decreasing interleukin (IL)-17, IL-6, and interferon-γ levels as well as increasing the level of IL-10 secreted by T-cells (Li et al., 2021). As for corneal diseases, the most common one is corneal epithelial injury, and iPSCs/MSC-Exos can also promote the regeneration of corneal epithelial cells by upregulating cyclin A and CDK2 (Wang et al., 2020b). Among the types of uveitis, most of which are autoimmune diseases, through reducing the pro-inflammatory cytokines, IFN-γ^+^CD4^+^ cells, and IL-17^+^CD4^+^ cells, MSC-Exos can alleviate ocular inflammation (Zhang et al., 2021). Taking choroidal neovascularization, a sticky neovascular-related retinal disease, as an example, stem cell EVs can improve the visual function and tissue structure by reducing the expression of vascular endothelial growth factor (VEGF) A (Yaghoubi et al., 2019). Diabetic retinopathy (DR), a major cause of blindness in the aged population, is a type of microangiopathy whose pathogenesis includes both occlusion and leakage of microvascular contents. Currently, the treatment of DR is mainly aimed at its prevention. MSC-Exos can downregulate the expression of high-mobility group box protein 1, NOD-like receptor thermal protein domain–associated protein 3, and nuclear factor kappa-light-chain-enhancer of activated B-cells/p65 protein to alleviate retinal vascular endothelial injury. Also, miR-222, contained in MSC-Exos, can act on retinal cells and regulate the expression of signal transducers and the transcriptional activator 5A protein, inhibit the formation of neovascularization in DR, and promote retinal regeneration (Xiong et al., 2021). Further, MSC-Exos can inhibit the induction of tumor necrosis factor (TNF)-α and reduce the inflammatory response in the retina (Nuzzi et al., 2020). Glaucoma and optic nerve–related diseases are characterized by optic lesions and, in this context, SC-EVs can downregulate Th1 and Th17, affecting the level of TNF-α, then upregulate pigment epithelial-derived factors and VEGF to reduce ocular inflammation and protect optic nerve cells (Mead et al., 2020). With the help of miRNA contained in exosomes, MSC-Exos can protect retinal ganglion cells (Mead and Tomarev, 2017; Mead et al., 2018) (Figure 3).

### 1.4 Therapeutic mechanism of stem cell-derived EVs for retinal degeneration

**FIGURE 4:**
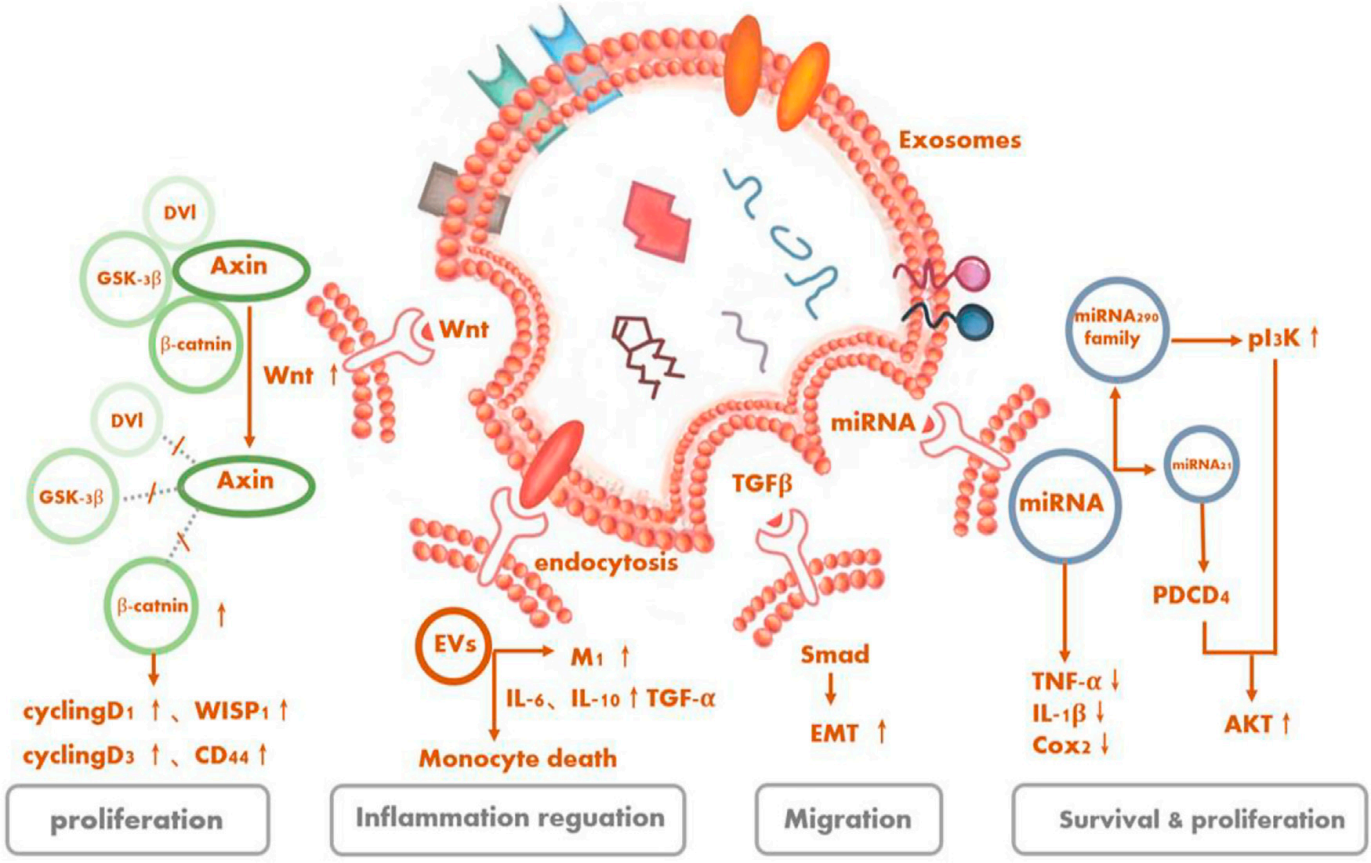
Signaling pathways as underlying potential mechanisms of stem cell-derived extracellular vesicles for retinal degeneration.

Figure 4Multiple studies have demonstrated in retinal degenerative diseases that the functions of tissue repair, cell protection, and so on of stem cells derived from EVs are not inferior and may even be better than the cell-replacement effects of stem cells because of the lower tumorigenicity rates of the former. Yet, the therapeutic mechanism of EVs, especially concerning what specific substances and through which exact pathway(s) exosomes exert their protective effects for treating RD, is still under investigation.

#### 1.4.1 Phosphoinositide 3-kinase (PI3k/Akt)

Serine/threonine kinase Akt, also known as protein kinase B, is the central node of many signaling downstream factors, including growth factors, cytokines, and other cellular-stimulation ligands. PI3K/Akt is a classical pathway that can mediate cell activities, such as cell survival and proliferation, and is activated by a variety of substances (Manning and Cantley, 2007). MSC-Exos can also regulate cellular activities of target cells through this pathway. It is recognized that oxidative stress and inflammation are involved in the development of RD. Especially, the production of reactive oxygen species can cause great damage to retinal cells (Jarrett and Boulton, 2012; Nita and Grzybowski, 2016; Yako et al., 2021). MSC-Exos contain five enzymes required for generating adenosine triphosphate (ATP) during glycolysis. CD73 on the surface can enhance the ATP-generation and antioxidant abilities of the cells by inducing phosphorylation of Akt and glycogen synthase kinase 3 (Arslan et al., 2013). The complex immune protein Toll-like receptor 4 (TLR4), a key target of biological metabolism, was initially thought to play a role in innate immunity, but subsequent studies have shown that it not only exists in MSCs but also has a protective effect on damaged nerve cells (Pradillo et al., 2009; Zeuner et al., 2015). MSC-EVs have the ability to affect the proliferation, migration, and differentiation of MSCs themselves and additionally reduce the inflammatory response of cells through the activation of the TLR4/PI3K/Akt signaling pathway (Zhao et al., 2019).

Liu et al. (2020) showed that ESCs co-cultured with RPE cells could alleviate the aging of RPE cells *in vitro* and promote their proliferation by activating the PIK3 pathway. Furthermore, ESC-EVs could promote the counter-differentiation of Müller cells into retinal precursors by inhibiting the expression of octamer-binding transcription factor 4 in retinal Müller cells, which contributes to the renewal of retinal cells (Ke et al., 2021). By activating signaling pathways (TGFb1, SMAD3, ID1, ID3 and PIK3CG, PDK1, and PLK1) mediated by transforming growth factor-β (TGF)-β and PI3K signaling factors, ESCs can downregulate essential effectors of cell senescence, such as p53, p21 (WAF1/CIP1), and p16 (INK4a), and upregulate cyclins, including cyclins A2, B1, and D1, thus promoting cell division and preventing cell cycle arrest mediated by cyclin-dependent kinase inhibitors like p21, thereby delaying the aging of RPE cells (Wang et al., 2020a).

#### 1.4.2 TGF-β1

TGF-β1, a member of the TGF-β superfamily, is closely related to cell apoptosis and has varied roles in cellular activities, like immunosuppression. Mothers against decapentaplegic (Smad) protein is a direct substrate of the TGF-β superfamily that can carry signals transmitted by TGF-β into the nucleus and affect the transcriptional regulation process in cells. Smad2 can be directly activated by TGF-β1. Activated TGF-β1 interacts with phosphorylated Smad2 to mediate cell life activities (Bakhshayesh et al., 2012; Frick et al., 2017).

The exosomes derived from MSCs possess the ability to ameliorate liver fibrosis by reducing TGF-β1 expression, inactivating the phosphorylation of Smad2, and reversing liver epithelial–mesenchymal transition (Li et al., 2013). MSC-EVs are capable of reversing chronic inflammation while being internalized by macrophages and tubular cells. What enriched in EVs like IL-4 and IL-10 progeny drive the pro-inflammatory (M1) macrophages’ shift to trophic (M2) macrophages. Moreover, EVs can decease several pro-inflammatory cytokines, such as TNF-α, IL-1β, IL-6, and monocyte chemoattractant protein (MCP)-1 (Mosser and Edwards, 2008; Eirin et al., 2017). Correspondingly, EVs secreted by the RPE can inhibit T-cell proliferation with or without stimulation of inflammation cytokines like IL-1β, IFN-c, and TN1F-α, albeit in different ways. Whether by inducing an immunoregulatory-phenotype monocyte, especially the classical phenotype, and upregulating TGF-β1 or diminishing the number of monocytes, all in all, EVs exert immunomodulatory effects to mitigate the possible harmful inflammatory response (Knickelbein et al., 2016).

#### 1.4.3 MCP-1

The nuclei of photoreceptor cells are involved in the formation of the outer nuclear layer (ONL). In RD, this structure is typically damaged, and damaged photoreceptor cells can lead to structural disorders and thickness changes in the inner nuclear layer and ONL in related retinal regions (Hartong et al., 2006; Huang et al., 2014). Lipopolysaccharide, a bacterial endotoxin, is considered to aggravate the expressions of IL-6, MCP-1, and miR-21, resulting in the impairment of RPE cells (Liu et al., 2019). Recently, MSC-Exos have been regarded to confer retinal protection activity by downregulating the messenger RNA expressions of MCP-1, TNF-α, and ICAM1 to blunt retinal inflammation, thereby reducing the aggregation of inflammatory cells like macrophages, improving the structure of the ONL, and protecting the photoreceptors (Yu et al., 2016).

#### 1.4.4 Wingless/integrated

Wnt is often involved in embryonic development and tumor growth, mainly by using paracrine or autocrine routes to transmit signals intercellularly or intracellularly. There are many classifications of Wnt signaling pathways, the most important of which is the Wnt/β-catenin signaling pathway. The proteins involved in Wnt signaling pathways are frequently overexpressed in cancer cells, contributing to active cell proliferation (Stewart, 2014).

The activation of a Wnt signaling pathway in RPE is one of the retinal self-protection mechanisms in retinal degenerative diseases, which could halt further photoreceptor degeneration and elevate retinal cell regeneration (Osakada et al., 2007; Yi et al., 2007). Some studies have recapitulated that the Wnt contained in MSC-Exos should act on targeted cells in an autocrine or paracrine manner and mediate the cell activity by stimulating a Wnt pathway (Zhang et al., 2015; Cui et al., 2017a; Shi et al., 2017; Rong et al., 2019). In addition, Wnt/β-catenin signaling was also suggested relative to the proliferation and repair of human iPSC-RPE (Cho et al., 2019).

#### 1.4.5 microRNAs

miRNAs are the non-coding single-stranded RNAs that regulate gene expression. As mentioned above, EVs can contain a variety of miRNAs (Mathivanan et al., 2012). The types and amounts of miRNAs in RPE-derived exosomes closely correlate with the occurrence of retinal tissue inflammation and degenerative diseases. The miRNAs contained in exosomes derived from stem cells can inhibit or promote the expression of related genes and regulate the activities of targeted cells by binding to certain genes.

##### 1.4.5.1 Mesenchymal stem cells

The miRNAs coated in MSC-Exos can alter target cell activities. In bone, MSC-Exos encapsulated miR-21 can inhibit the expression of the *PTEN* gene, thus activating the PI3K/Akt signal transduction pathway, increasing the threshold for apoptosis of nucleus pulposus cells (Pittenger et al., 2019). In oncology, MSC-Exos have been trialed as delivery vectors for transporting certain miRNAs to the targeted cells, further regulating the genes in resident cells (83–86). Deng et al. (2020) injected MSCs and MSC-Exos into the vitreous cavities of mice and confirmed that they had a long-term and lasting protective effect on preventing the loss of retinal photoreceptors and retinal function. Even a single injection could have a protective effect for up to 4–8 weeks. However, when the MSCs were pretreated with the EV-secretion inhibitor GW4869, their protective effect was inhibited. Next, the investigators identified the therapeutic effects of MSC-Exos, whose anti-apoptotic effects are mediated by miR-21, which targeted programmed cell death 4. Through this process, the thickness of ONL could be increased, which should be regarded as having a protective effect on retinal photoreceptor cells as mentioned earlier.

##### 1.4.5.2 Neural progenitor cells

Under normal physiological conditions, miR-21 carried by RPE-derived exosomes could lead to the occurrence of retinal neuro-inflammation and accelerated RD progression by altering the function of the subretinal microglia (Morris et al., 2020), while NPC-derived exosomes play a role in delivering miR-21 from parent cells to targeted cells. The function and ability of exosomes is affected by the amounts of miRNAs they contain. Under specific conditions, miR-21 can activate Akt and Wnt signaling pathways, then achieve protective effects by promoting the generation of neurons (Ma et al., 2019).

Royal College of Surgeons (RCS) rats with *MERTK* gene mutations constitute an animal model of RD in which the RPE has lost the ability to phagocytose the outer segment of photoreceptor cells, resulting in an accumulation of falling debris of the outer segment, which blocks nutritional support from the choroid to photoreceptor cells, consequently leading to the progressive death of ONL cells and a reduction in ONL thickness. In RCS rats, microglia activation causes the release of pro-inflammatory factors, triggering impairments in the retinal ONL and photoreceptors. It has been recognized that NPCs could be obtained from the human retina and mouse subventricular zone. After being injected into the subretinal cavity of RD model RCS rats, microglia can internalize mouse NPC-derived exosomes, and the miRNA contained in mouse NPC-derived exosomes can inhibit the microglia activation induced by lipopolysaccharide; reduce the release of pro-inflammatory factors, such as cyclooxygenase-2, TNF-α, and IL-1β; participate in the downregulation of multiple inflammatory pathways, including mitogen-activated protein kinase, IL-17, nuclear factor kappa-light-chain-enhancer of activated B-cells, and TNF; prevent the injury of ONL cells; and reduce the apoptosis of photoreceptors (Bian et al., 2020).

##### 1.4.5.3 Embryonic stem cells

ESC-derived exosomes also release miRNA to targeted cells. For example, ESC-derived exosomes can be transferred to cardiac precursor cells (CPCs) to release members of their packed miR-290 family—especially miR-294, which is not normally expressed in adult cells or organs—to phosphorylate Akt in CPCs, regulate the cell cycle, promote cell proliferation and survival, and assist in increasing the number as well as function of c-kit^+^ CPCs, thereby contributing to the development of new cardiomyocytes and enhanced myocardial viability (Cui et al., 2017b).

## 2 Concluding remarks and future perspectives

Human MSCs (hMSCs) were first utilized for clinical trials in 1993; since then, >10,000 people have been treated with autologous or allogeneic hMSCs (Lazarus et al., 1995; Pittenger et al., 2019). In traditional experiments, many researchers have successfully transplanted stem cell-derived induced cells, though the therapeutic effect varied and they failed to find the transplanted cells after a short period of time in the experimental subjects. In 2007, Yoshitaka et al. (Iso et al., 2007) recognized that, when hMSCs were intravenously administered in a myocardial infarction mouse model, the cardiac function of the mice injected with hMSCs was improved compared to the control group. Nevertheless, no evidence supported hMSC engraftment in the heart; instead, multiple protective factors secreted by the engrafted hMSCs were detected, and the investigators assumed that it was the paracrine effects or secreted factors of hMSCs that ameliorated cardiac dysfunction. These findings prompted researchers to consider if stem cells’ therapeutic effects are really achieved by replacing lost and/or defected cells in diseased tissues; researchers also questioned the mechanism underlying the therapeutic effects stem cell replacement. In an effort to elucidate the mechanisms, many researchers co-cultured stem cells with impaired experimental cells. Interestingly, although the stem cells are not directly in contact with the experimental cells, an effective therapeutic effect still existed. It was hypothesized that the therapeutic effect of stem cells appears to be attributable to the substances they secreted, rather the engraftment, differentiation, or cell fusion of stem cells.

EVs, as their name suggests, can be regarded as a type of vehicle with strong transportation capacity. As mentioned above, EVs can pack in a variety of substances and have their own characteristic marks; without stopping here, it is intelligent to change their characteristics and materials contained according to the different microenvironments around them and their targeted cells. Therefore, it is quite prospective to use the vehicle’s power to transport the substances we design.

Considering stem cell therapy for RD, most of the current clinical trials or pre-clinical trials still engraft the RPE derived from stem cells. The sources of stem cells can be divided into autologous and allogeneic types. Autologous stem cells have less immunogenicity; thus, the possibility of their rejection is lower than that of allogeneic stem cells, but it is not that convenient to obtain the initial patient tissue in this context. Correspondingly, the obstacle in allogeneic stem cell transplantation is the immunosuppressive effects. Usually, surgical methods include vitreous cavity stem cell injection or transplantation of engraft-induced RPE grafts or RPE cells into the subretinal cavity. Subretinal injection of induced RPE cells can be divided into two methods: suspension and with a scaffold (Siqueira et al., 2011; Schwartz et al., 2015; Oner et al., 2016; Mandai et al., 2017; da Cruz et al., 2018; Kashani et al., 2018; Mehat et al., 2018).

In recent years, more and more observations have validated the idea that the essence of the therapeutic effect of stem cells probably arises from the exosomes released by said stem cells. In experimental research, exosome transplantation is considered to achieve the same therapeutic effect as stem cell transplantation and circumvents the limitations associated with direct cell administration so that the therapeutic safety can be further improved, thereby making this approach more conducive to clinical application. As mentioned above, stem cells themselves have multipotency or even pluripotency, which makes it difficult to control their differentiation direction or differentiation product(s), especially when transplanting stem cells in a complex microenvironment like the retina. Furthermore, stem cell acquisition involves unique ethical issues, thus facing more barriers than expected. Nonetheless, as a paracrine product of stem cells, exosomes are easier to sterilize and preserve after acquisition with a more stable biological profile. In the future, exosomes will also be easier to commercialize for extensive use. However, an issue that should be considered is the variations in the quality and physiological function of EVs depending on the age of the parent cell. EVs from senescent MSCs probably lose their treatment efficacy (Ahmadi et al., 2021). In addition, because of the characteristics of exosomes, researchers have found that exosomes can be customized to establish novel therapeutic strategies. In oncology, exosomes containing certain miRNAs are modified by specific surface proteins to make them chemotactic to targeted cells for boosting apoptosis, diminishing proliferation, preventing migration, and monitoring exosome-mediated autophagy (Hassanpour et al., 2021; Nie et al., 2020; Kim et al., 2021).

In sum, in the future of treatment for RD, if the mechanisms underlying RPE or photoreceptor cell impairment are deciphered, we can design and edit upstream or downstream components at the gene level by using modified exosomes, thus blocking the RD damage pathway to ameliorate cell damage sequentially. This method shows a promising chance to become an effective and safe way to save patients’ vision.
